# Diverse Mechanisms of BRAF Inhibitor Resistance in Melanoma Identified in Clinical and Preclinical Studies

**DOI:** 10.3389/fonc.2019.00268

**Published:** 2019-04-17

**Authors:** Stephen A. Luebker, Scott A. Koepsell

**Affiliations:** Department of Pathology and Microbiology, University of Nebraska Medical Center, Omaha, NE, United States

**Keywords:** melanoma, BRAF inhibitor, vemurafenib, dabrafenib, cell line, drug resistance, metabolism, invasion

## Abstract

BRAF inhibitor therapy may provide profound initial tumor regression in metastatic melanoma with BRAF V600 mutations, but treatment resistance often leads to disease progression. A multi-center analysis of BRAF inhibitor resistant patient tissue samples detected genomic changes after disease progression including multiple secondary mutations in the MAPK/Erk signaling pathway, mutant BRAF copy number gains, and BRAF alternative splicing as the predominant putative mechanisms of resistance, but 41.7% of samples had no known resistance drivers. *In vitro* models of BRAF inhibitor resistance have been developed under a wide variety of experimental conditions to investigate unknown drivers of resistance. Several *in vitro* models developed genetic alterations observed in patient tissue, but others modulate the response to BRAF inhibitors through increased expression of receptor tyrosine kinases. Both secondary genetic alterations and expression changes in receptor tyrosine kinases may increase activation of MAPK/Erk signaling in the presence of BRAF inhibitors as well as activate PI3K/Akt signaling to support continued growth. Melanoma cells that develop resistance *in vitro* may have increased dependence on serine or glutamine metabolism and have increased cell motility and metastatic capacity. Future studies of BRAF inhibitor resistance *in vitro* would benefit from adhering to experimental parameters that reflect development of BRAF inhibitor resistance in patients through using multiple cell lines, fully characterizing the dosing strategy, and reporting the fold change in drug sensitivity.

## Introduction

Melanoma makes up 6% of estimated new cancer cases in men and 4% in women, and incidence has been increasing since 1975 (1). BRAF mutations occur in more than 50% of cutaneous melanomas, and BRAF V600E occurs most frequently, which confers constitutive monomeric activation of BRAF kinase activity (2, 3). The identification of oncogenic BRAF signaling increased interest in targeted inhibitors toward mutant BRAF variants, and the FDA has approved two targeted BRAF inhibitors, vemurafenib in 2011 and dabrafenib in 2013, for treatment of non-resectable BRAF V600E/K mutant melanoma. Despite the rapid response and short-term increases in patient survival, resistance to BRAF inhibition persists. In 2017, combination therapy of dabrafenib plus the MEK inhibitor, trametinib was FDA approved for treatment of melanoma to forestall the development of BRAF inhibitor resistance. This review summarizes the potential events driving BRAF inhibitor resistance detected in patient tissue and contrasts them with *in vitro* studies of BRAF inhibitor resistance through comparison of methods and results.

### BRAF Inhibitor Resistance in Patients With Melanoma

Phase-3 clinical trials of vemurafenib treatment for BRAF V600E/K melanoma demonstrated improvements in median progression-free survival relative to dacarbazine (6.9 months vs. 1.6 months) and increased median overall survival (13.6 vs. 9.7 months) (4). Phase-3 clinical trials of dabrafenib treatment for BRAFV600E melanoma observed improvements in median progression free survival relative to dacarbazine (5.1 vs. 2.7 months) (5). Phase-3 clinical trials of dabrafenib and trametinib combination therapy vs. dabrafenib alone found increased median progression-free survival (11.1 vs. 8.8 months) and increased median overall survival (25.1 vs. 18.7 months) (6). Treatment with BRAF/MEK inhibitors often provides remarkable disease regression initially, but resistance to therapy frequently develops within 12 months as indicated by median progression-free survival.

**Figure 1:**
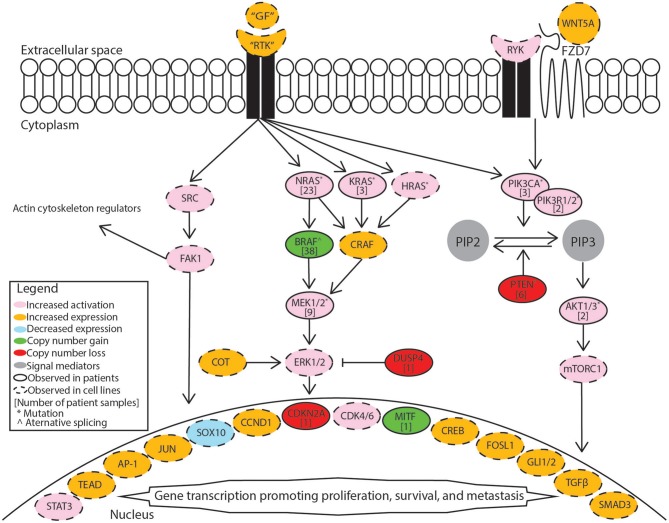
Mechanisms supporting BRAF inhibitor resistance in melanoma. Receptor tyrosine kinases (RTK) include AXL receptor tyrosine kinase (AXL), epidermal growth factor receptor (EGFR), fibroblast growth factor receptor 1 (FGFR1), fibroblast growth factor receptor 3 (FGFR3), platelet-derived growth factor receptor beta (PDGFRB), MET proto-oncogene receptor tyrosine kinase (MET), and KIT proto-oncogene receptor tyrosine kinase (KIT). Growth factors (GF) correspond to the specific receptor tyrosine kinase. The MAPK/Erk pathway includes the Ras GTPases (N/K/HRAS), Serine/threonine-protein kinase B-raf (BRAF), RAF proto-oncogene serine/threonine-protein kinase (CRAF), mitogen-activated and extracellular signal-regulated kinase kinase 1 or 2 (MEK1/2), extracellular signal-regulated kinase 1 or 2 (ERK1/2), cancer Osaka thyroid (COT), and dual specificity protein phosphatase 4 (DUSP4). The PI3K/Akt pathway includes phosphatidylinositol-4,5-bisphosphate 3-kinase catalytic subunit alpha (PIK3CA), phosphatidylinositol 3-kinase regulatory subunit 1 or 2 (PIK3R1/2), phosphatidylinositol 4,5-bisphosphate (PIP2), phosphatidylinositol 3,4,5-trisphosphate (PIP3), phosphatase and tensin homolog (PTEN), AKT serine/threonine kinase 1 or 2 (AKT1/2), mammalian target of rapamycin complex 1 (mTORC1). Src signaling factors include SRC proto-oncogene non-receptor tyrosine kinase (SRC) and focal adhesion kinase 1 (FAK1). Transcription factors include signal transducer and activator of transcription 3 (STAT3), TEA domain transcription factor protein family (TEAD), activator protein 1 complex (AP-1), Jun proto-oncogene AP-1 transcription factor subunit (JUN), SRY-box 10 (SOX10), melanocyte inducing transcription factor (MITF), cyclic AMP responsive element binding protein family (CREB), FOS like 1 AP-1 transcription factor subunit (FOSL1), GLI family zinc finger 1 or 2 (GLI1/2), transforming growth factor beta (TGFβ), SMAD family member 3 (SMAD3). Cell cycle regulators included cyclin D1 (CCND1), cyclin dependent kinase 4 or 6 (CDK4/6). Non-canonical Wnt signaling mediators include receptor like tyrosine kinase (RYK), frizzled class receptor 7 (FZD7), and Wnt family member 5A (WNT5A).

BRAF inhibitor resistance in melanoma is supported through recovery of MAPK/Erk signaling or activation of PI3K/Akt signaling. These pathways may be activated through mutations, copy-number alterations, or changes in expression. A summary diagram including these signaling pathways and a breakdown of common alterations supporting BRAF inhibitor resistance are illustrated in Figure 1. A multi-center analysis of BRAF inhibitor resistance combining three comprehensive genome sequencing studies of pre-treatment and post-progression cases of melanoma identified resistance driving events in 58.3% (77/132) of samples obtained from 100 individuals, but failed to identify any known mechanism of resistance in the remaining 41.7% of samples (7). Johnson et al. provide a complete breakdown of the frequency of the resistance mechanisms within this combined data set. Multiple resistance mechanisms were observed within individual samples and unique resistance mechanisms were observed between samples from the same patient. BRAF amplification and alternative splicing were observed most frequently followed by NRAS mutations and MEK1/2 mutations. Mutations in the PI3K/Akt pathway are less frequently observed in patient samples. Despite increased median progression-free survival when treating patients with dabrafenib plus trametinib relative to dabrafenib alone, treatment resistance still develops. Patients treated with dabrafenib/trametinib combination therapy developed alterations in the same genes that support single-agent resistance including MEK1/2 mutations, BRAF amplification, BRAF alternative splicing, and NRAS mutations between pre-treatment and post-progression samples (8, 9). Clinical studies of BRAF inhibitor resistance leave an incomplete picture of the diverse set of mechanisms supporting BRAF inhibitor resistance. This review summarizes recent studies in which BRAF inhibitor resistance was induced stochastically in cell lines via prolonged exposure to a BRAF inhibitor. Major mechanisms identified in these studies are included in Figure 1 and discussed in more detail in this review.

### BRAF Inhibitor Resistance in Melanoma Cell Lines

#### Receptor Tyrosine Kinase Expression

Receptor tyrosine kinases may act as upstream activators of MAPK/Erk signaling, and increased expression in BRAF inhibitor resistant cells has been described in multiple studies. Shaffer et al. demonstrated that resistance to BRAF inhibitors in WM989 and WM983B cells occurs through non-heritable, transient expression of multiple resistance-associated genes including receptors like AXL receptor tyrosine kinase (AXL), epidermal growth factor receptor (EGFR), fibroblast growth factor receptor 1 (FGFR1), and platelet-derived growth factor receptor beta (PDGFRB) among others (10). Other studies have detected expression changes in these genes but do not point to a single pattern of expression change. Nazarian et al. demonstrated that increased expression of PDGFRB conferred resistance to M229 and M238 cells, but Jazirehi et al. found that resistant M238 cells had increased expression of EGFR and decreased expression of PDGFRB (11, 12). Shao et al. found resistant WM793 and M238 cells both had increased PDGFRB but decreased Insulin-like growth factor 1 receptor (IGF1R) expression (13). Increased PDGFRB expression has also been described in resistant A375 cells (14). In two other studies using A375 cells, increased expression of fibroblast growth factor receptor 3 (FGFR3) expression was associated with BRAF inhibitor resistance (15, 16). Resistant A375 cells have also been shown to increase expression of IGF1R while resistant SKMEL28 cells increased expression of PDGFRB (17). In a separate study, resistant SKMEL28 cells had increased expression of both EGFR and PDGFRB (18). Jazirehi et al. found that resistant M249 cells had increased expression of EGFR, KIT proto-oncogene receptor tyrosine kinase (KIT), MET proto-oncogene receptor tyrosine kinase (MET), and PDGFRB with decreased IGF1R (11). Resistance-associated gene expression may occur through loss of SOX10 expression and gain of JUN, AP-1, and TEAD transcription factor activity (10). EGFR expression may be regulated through MITF expression, but both increased and decreased MITF expression have been observed in BRAF inhibitor resistant cell lines (19, 20). Sun et al. demonstrated that miR-7 was significantly downregulated in resistant A375 and MEL-CV cells, and exogenous expression could reduce resistance with EGFR, IGF1R, CRAF, and AXL as potential targets (21). Overall, changes in growth factor expression are inconsistent between studies using the same cell lines. Increased expression of any growth factor receptor that activates MAPK/Erk may potentially drive resistance in melanoma.

#### Secondary MAPK/Erk Mutations

In addition to upstream activation of MAPK/Erk through receptor tyrosine kinases, increased MAPK/Erk signaling may be achieved through direct alteration to members of the RAS/RAF/MEK/Erk signaling cascade. Secondary BRAF mutations and alternative BRAF splicing have been shown to induce vemurafenib resistance in multiple cell lines (19, 22). In a patient derived xenograft model, increased BRAF V600E expression sustained resistance, and cells demonstrated drug-dependence for continued proliferation (23). Resistant tumors derived from 1205LU cells in a mouse xenograft model contained distinct alternative BRAF splicing events in two tumors and HRAS Q61K mutation in one tumor (24). Other alterations within RAS/RAF/MEK/Erk cascade have been observed in SKMEL28, A375, COLO829, and M249 cells, including COT overexpression and NRAS Q61K mutation (12, 17). Dabrafenib resistant A375 and MEL-RMU cells were found to have mutations in MEK1 and NRAS as previously described in vemurafenib resistant cells (25, 26). NRAS mutations may also make cell lines cross-resistant to MEK inhibitors due to elevated PI3K/Akt signaling (27). Resistant A375 cells were found to have an NRAS G13R mutation, high expression of CRAF, and increased Akt phosphorylation (28). Resistant A375 cells with a KRAS K117N also had elevated expression of CRAF and activation of Akt (29). Resistant M249 cells and M376 cells with secondary NRAS mutations had increased Akt activation (30). Resistant WM793 cells with secondary NRAS Q61K mutation require CRAF expression and SHOC2 scaffold protein to re-activate MAPK/Erk (31). *In vitro* models of BRAF inhibitor resistance indicate that secondary mutations may support increased activation of MAPK/Erk in the presence of inhibitor or support sustained growth through activation of PI3K/Akt signaling.

#### Alternative Resistance Pathways

Downstream effectors of PI3K/Akt activation promote survival of resistant cells. PI3K/Akt activation upregulates AEBP1 through increased CREB binding, and increased AEBP1 leads to IκBα degradation and NF-κB activation (32). A375, SKMEL28, and WM239 cells resistant to either dabrafenib or vemurafenib all had increased expression of Mcl-1 relative to their pre-treatment counterparts, which promotes cell survival through inhibition of apoptosis, and Mcl-1 expression may be regulated by STAT, cAMP, and NF-κB binding sites (33). Growth factor receptors may also cross activate PI3K/Akt separately or in addition to MAPK/Erk activation. Resistance induced in SKMEL28 cells increased expression of EGFR and activated Akt (34). Resistant LM17 cells had increased IGF1R expression as well as increased Akt phosphorylation (35). Increased expression of WNT5A in A375 and MEL-264 was correlated with increased phosphorylation of Akt and activation of RYK and FZD7 receptors supporting non-canonical Wnt signaling (36). PI3K/Akt activation in multiple BRAF inhibitor resistant melanoma cell lines also up-regulates of FOSL1, which drives secretion of multiple factors from tumor cells that support surrounding tumor growth (37). Melanoma cells may support the resistance of surrounding cells in addition to other stromal cells. Hepatocyte growth factor (HGF) secretion by surrounding stromal cells in co-culture supports tumor growth in the presence of BRAF inhibitors through activation of the MET receptor tyrosine kinase and downstream MAPK/Erk activation (38). The adaptive resistance of melanoma cells may be supported through both neighboring cancer and non-cancer cells. MAPK/Erk signaling and/or PI3K/Akt signaling may be activated in BRAF inhibitor resistant cells frequently through common mechanisms.

### Phenotypic Changes in BRAF Inhibitor Resistant Cell Lines

#### Increased Motility and Invasion

Resistant cell lines acquire a more invasive phenotype characterized by increased cell motility and metastatic capacity. Multiple studies have noted increased invasive capacity of BRAF inhibitor resistant melanoma cell lines, and recent proteomic studies of melanoma cell lines before and after developing BRAF inhibitor resistance have specifically characterized differences in kinase expression and changes in phosphorylation. Quantitative phosphoproteomics of vemurafenib resistant LM-MEL-28 cells demonstrated increased activation of MAPK/Erk signaling and de-phosphorylation of key cytoskeletal regulators (39). Activity-based protein profiling of kinases in WM164, WM793, A375, and 1205LU cells detected increased ATP uptake by FAK1, SLK, LYN, PRKDC, and KCC2D, but overall changes between cell lines showed differences in differential phosphorylation (40). Phospho-array analysis and quantitative phosphoproteomics identified increased EGFR phosphorylation in vemurafenib resistant A375 and COLO829 cell lines leading to Src family kinase phosphorylation and STAT3 activation, which was associated with increased invasion and phosphorylation of cytoskeletal proteins (41). The increase in cytoskeletal remodeling also has downstream effects in cell signaling. For example, actin remodeling has been shown to increase YAP/TAZ nuclear localization in BRAF inhibitor resistant melanoma cells, and YAP/TAZ nuclear localization increases expression of EGFR, AKT, and MYC (42). The expression of receptor tyrosine kinases is associated with the invasive behavior of melanoma cell lines through increased metalloprotease expression. EGFR signaling was found to drive resistance in SKMEL28 cells, and resistance was also associated with upregulation of MMP2 and downregulation of the MMP regulator, TIMP2 (43). Increased expression of EGFR in SKMEL28 cells was also correlated with increased activation of Non-canonical Hedgehog Signaling (GLI1, GLI2, TGFβ, and SMAD3), and inhibition of GLI1 and GLI2 increased vemurafenib sensitivity while decreasing invasiveness (44). Dabrafenib resistant A375 cells had increased expression of epithelial to mesenchymal transition markers including CD90 and decreased expression of E-cadherin with increased cell motility (45). A separate study of dabrafenib resistant A375 cells also detected increased secretion of VEGFA and MMP9, which was associated with increased invasiveness (46).

#### Metabolism

Alterations in the metabolism of BRAF inhibitor resistant cells have also been described, including increased dependence on serine or glutamine. Vemurafenib resistant SKMEL28 were dependent on serine metabolism, and knockout of PHGDH or depletion of serine in the media reduced viability of resistant cells (47). Additionally, vemurafenib resistant M229 and M249 cells or vemurafenib/selumetinib dual treatment resistant M249 cells had increased glutamine uptake and were dependent on glutamine for survival independently of the underlying mechanism of resistance (48). More complex metabolic reprogramming may occur during the development of BRAF inhibitor resistance. Gene set enrichment of KEGG pathways using quantitative phosphoproteomic analysis of vemurafenib resistant LM-MEL-28 cells detected enrichment in DNA replication and cell cycle but decreases in glycolysis/gluconeogenesis, fatty acid metabolism, valine/leucine/isoleucine degradation, pyruvate metabolism, and tryptophan metabolism (39).

### Future Directions of *in vitro* Research

McDermott et al. have recently published a general review of important considerations for developing *in vitro* resistance to targeted inhibitors and chemotherapeutic agents in cancer cell lines (49). Important considerations for *in vitro* models of BRAF inhibitor resistance in melanoma cell lines include choice of cell line, dosing strategy, and resistant cell selection criteria. Examples of current methods that have been applied to A375 cells are summarized in Table 1. This review focuses on studies that develop resistant cell lines through drug treatment and excludes studies of primary resistance or genetically induced resistance.

**Table 1:** *In vitro* BRAF inhibitor resistance in A375 melanoma cells.

**References**	**Drug details**	**Treatment strategy**	**Dose (μM)**	**Clonal selection**	**Time to resistance**	**IC50 (μM)**	**Resistance driver**	**Pathway reactivation**
Cordaro et al. (45)	Dabrafenib	Increasing continuous	NA	None	4 weeks	0.0095–110.5	Undetermined	Undetermined
Greger et al. (25)	Dabrafenib	Increasing continuous	up to 1.6	Single cell derived clones	NA	0.028 to >10	MEK1 K59del	pErk
Caporali et al. (46)	Dabrafenib	Increasing continuous	0.001–1.5	None	4 months	0.001041 to >10	Undetermined	pErk and pAkt
Zeiderman et al. (50)	Vemurafenib	Continuous	1	None	1 year	NA	Undetermined	Undetermined
Smyth et al. (14)	Vemurafenib	Continuous	2	None	3 weeks	0.087 to > 10	Undetermined	Undetermined
Jameson et al. (17)	Vemurafenib	Continuous	1	Colonies by limiting dilution	4-6 weeks	0.287–13.47	PDGFRB overexpression	Undetermined
Graziani et al. (28)	Vemurafenib	Increasing continuous	up to 2.5	None	3 months	1.47–20.37	NRAS G13R and CRAF overexpression	pErk and pAkt
Anastas et al. (36)	Vemurafenib	Increasing continuous	2	None	10 weeks	NA	WNT5A overexpression	pAkt
Yadav et al. (15)	Vemurafenib	Increasing continuous	0.2–2	None	4 months	0.085–4.8	FGFR3 phosphorylation	pErk
Girotti et al. (41)	Vemurafenib	Increasing continuous	0.1–2	None	2 months	0.155–5.76	EGFR phosphorylation	pErk and pAkt
Ji et al. (19)	Vemurafenib	Increasing continuous	0.5–10	None	NA	>10-fold	BRAF splicing	pErk and pAkt
Muller et al. (20)	Vemurafenib	Increasing continuous	up to 3	None	NA	NA	MITF loss	pErk
Sharma et al. (40)	Vemurafenib	Continuous	2.5	None	3 months	NA	Undetermined	Undetermined
Wang et al. (18)	Vemurafenib	Increasing continuous	up to 2.5	None	3 months	0.57–28.9	PDGFRB overexpression	pERK and pAKT
Sun et al. (21)	Vemurafenib	Increasing continuous	up to 2	None	6 months	NA	EGFR, IGF1R, and CRAF overexpression	pERK
Obenauf et al. (37)	Vemurafenib	Increasing continuous	1–3	Low density seeding colonies	8 weeks	NA	EGFR and MET overexpression	pERK
Su et al. (29)	Vemurafenib	Increasing continuous	NA	None	3 months	86–119-fold	KRAS K117N	pERK and pAKT
Fofaria et al. (33)	Vemurafenib	Pulsed	0.2–10	None	1 year	0.1–3	Undetermined	pERK

#### Selecting Cell Lines

The first major consideration in this type of model is the degree of heterogeneity between cells. There is a great deal of cell-to-cell heterogeneity in melanoma *in vivo* (51). Sub-clones may harbor mutations conferring primary resistance to BRAF inhibitors. Selection of single-cell derived clones may reduce the heterogeneity observed within a single cell line. Studies show that there are genetic differences between cell lines and tumors *in vivo*, and only a few cell lines are most frequently used (52–56). *In vitro* resistance studies would benefit from using multiple cell lines to compare resistance mechanisms and potential novel combination therapy outcomes. The use of multiple cell lines also helps verify findings by highlighting mechanisms observed across cells types as opposed to findings that are specific only to that clone or test system.

#### Treatment Strategy

The treatment strategy employed to induce resistance in cell lines *in vitro* may or may not represent how the drug is administered clinically. Vemurafenib is administered as 960 mg tablets twice daily and reaches an average maximum plasma concentration of 4.8 ± 3.34 μg/ml after 8 h and 61.4 ± 22.76 μg/ml after 168 h with a half-life of 34.1 ± 19.66 h (57). Dabrafenib is administered as 150 mg oral tablets twice daily and reaches an average maximum plasma concentration of 986 ng/ml in a median 2 h with a half-life of 5 h (58). Both dabrafenib and vemurafenib quickly reach a high plasma concentration and have long half-lives, which would be best represented by continuously treating cells to develop resistance. Fofaria et al. employed a pulsed treatment strategy, which includes a treatment window followed by a recovery period, to generate vemurafenib resistant cell lines (33). A pulsed treatment strategy does not reflect how the drug is administered clinically. However, it has been shown that lower vemurafenib plasma concentration was significantly associated with higher likelihood of tumor progression, and patients had high inter-individual variability in vemurafenib plasma concentration (13.0–109.8 μg/ml) (59). Others have noted that the melanoma cell lines may become dependent on the presence of the BRAF inhibitor for continued growth and continuous treatment is often required, which may potentially be mitigated through a pulsed treatment method (13, 28, 34, 60). Mechanisms regulating development of resistance in each type of model may be different, and clear distinctions should be made between which type of model was employed. Data obtained from studies that use drug exposure methods never observed in patients should be interpreted with caution.

#### Defining Resistance

There is no standard for defining when a cell line is drug resistant. The selection criteria used to define treatment resistance critically influences results. Ideally the fold change in drug sensitivity should be reported along with the duration of drug exposure. Treatment durations for studies of A375 cells range from 6 days to 1 year of treatment, and fold change in drug sensitivity ranges from 3x to more than 100x (Table 1). Correlation to drug levels observed in patients should also be considered. Care must also be taken when reporting drug sensitivity since common colorimetric assays may not be accurate or reproducible due to variations in growth rate; a cell counting based method should be employed when possible (61, 62). Multiple studies have observed changes in cell line growth rate after developing treatment resistance, which may be dependent on the presence of drug (13, 34, 47, 60). Growth rate changes may confound the measurement of drug sensitivity between treatment resistant and pre-treatment cells.

## Conclusion

Although treatment with BRAF inhibitors provides rapid response in most patients, treatment resistance persists. The few clinical studies of BRAF inhibitor resistance in patients indicate that genetic alterations that activate MAPK/Erk make up half of resistance mechanisms. Preclinical studies of BRAF inhibitor resistance in melanoma support the mechanisms observed in patients and indicate that the development of resistance is more complex than single mutations. *In vitro* models may be very helpful in studying mechanisms in the other half of patients with no known genetic driver of BRAF inhibitor resistance. Overall, BRAF inhibitor resistance depends on oncogenic signaling through reactivation of MAPK/Erk or activation of PI3K/Akt, which may be acquired by directly affecting genes in each pathway, by upregulation of receptor tyrosine kinases, or by affecting downstream signaling. BRAF inhibitor resistance increases invasiveness through changes in phosphorylation actin cytoskeleton regulators and increased extracellular matrix metalloprotease expression. Resistant cells have also been shown to undergo metabolic reprogramming characterized by increased glutamine or serine dependence. A375 cells have been used to model BRAF inhibitor resistance across multiple studies, but the methods and conclusions vary. To improve preclinical *in vitro* research, future studies of BRAF inhibitor resistance in melanoma should include multiple cell lines, consider a continuous-dose treatment strategy, and report drug sensitivity in order to facilitate better comparison across studies.

## Author Contributions

All authors listed have made a substantial, direct and intellectual contribution to the work, and approved it for publication.

### Conflict of Interest Statement

The authors declare that the research was conducted in the absence of any commercial or financial relationships that could be construed as a potential conflict of interest.

## References

[1] Siegel RL Miller KD Jemal A Cancer statistics, 2018. CA Cancer J Clin. (2018) 68:7–30. 10.3322/caac.21442 29313949

[2] Zhang T Dutton-Regester K Brown KM Hayward NK. The genomic landscape of cutaneous melanoma. Pigment Cell Melanoma Res. (2016) 29:266–83. 10.1111/pcmr.12459 26833684

[3] Menzies AM Haydu LE Visintin L Carlino MS Howle JR Thompson JF. Distinguishing clinicopathologic features of patients with V600E and V600K BRAF-mutant metastatic melanoma. Clin Cancer Res. (2012) 18:3242–9. 10.1158/1078-0432.CCR-12-0052 22535154

[4] McArthur GA Chapman PB Robert C Larkin J Haanen JB Dummer R. Safety and efficacy of vemurafenib in BRAF(V600E) and BRAF(V600K) mutation-positive melanoma (BRIM-3): extended follow-up of a phase 3, randomised, open-label study. Lancet Oncol. (2014) 15:323–32. 10.1016/S1470-2045(14)70012-9 24508103 PMC4382632

[5] Hauschild A Grob JJ Demidov LV Jouary T Gutzmer R Millward M. Dabrafenib in BRAF-mutated metastatic melanoma: a multicentre, open-label, phase 3 randomised controlled trial. Lancet. (2012) 380:358–65. 10.1016/S0140-6736(12)60868-X 22735384

[6] Long GV Stroyakovskiy D Gogas H Levchenko E de Braud F Larkin J. Dabrafenib and trametinib versus dabrafenib and placebo for Val600 BRAF-mutant melanoma: a multicentre, double-blind, phase 3 randomised controlled trial. Lancet. (2015) 386:444–51. 10.1016/S0140-6736(15)60898-4 26037941

[7] Johnson DB Menzies AM Zimmer L Eroglu Z Ye F Zhao S. Acquired BRAF inhibitor resistance: a multicenter meta-analysis of the spectrum and frequencies, clinical behaviour, and phenotypic associations of resistance mechanisms. Eur J Cancer. (2015) 51:2792–9. 10.1016/j.ejca.2015.08.022 26608120 PMC4666799

[8] Wagle N Van Allen EM Treacy DJ Frederick DT Cooper ZA Taylor-Weiner A. MAP kinase pathway alterations in BRAF-mutant melanoma patients with acquired resistance to combined RAF/MEK inhibition. Cancer Discov. (2014) 4:61–8. 10.1158/2159-8290.CD-13-0631 24265154 PMC3947296

[9] Long GV Fung C Menzies AM Pupo GM Carlino MS Hyman J. Increased MAPK reactivation in early resistance to dabrafenib/trametinib combination therapy of BRAF-mutant metastatic melanoma. Nat Commun. (2014) 5:5694. 10.1038/ncomms6694 25452114

[10] Shaffer SM Dunagin MC Torborg SR Torre EA Emert B Krepler C. Rare cell variability and drug-induced reprogramming as a mode of cancer drug resistance. Nature. (2017) 546:431–5. 10.1038/nature22794 28607484 PMC5542814

[11] Jazirehi AR Nazarian R Torres-Collado AX Economou JS. Aberrant apoptotic machinery confers melanoma dual resistance to BRAF(V600E) inhibitor and immune effector cells: immunosensitization by a histone deacetylase inhibitor. Am J Clin Exp Immunol. (2014) 3:43–56. 24660121 PMC3960761

[12] Nazarian R Shi H Wang Q Kong X Koya RC Lee H Melanomas acquire resistance to B-RAF(V600E) inhibition by RTK or N-RAS upregulation. Nature. (2010) 468:973–7. 10.1038/nature09626 21107323 PMC3143360

[13] Shao Y Aplin AE. BH3-only protein silencing contributes to acquired resistance to PLX4720 in human melanoma. Cell Death Differ. (2012) 19:2029–39. 10.1038/cdd.2012.94 22858545 PMC3504716

[14] Smyth T Paraiso KHT Hearn K Rodriguez-Lopez AM Munck JM Haarberg HE. Inhibition of HSP90 by AT13387 delays the emergence of resistance to BRAF inhibitors and overcomes resistance to dual BRAF and MEK inhibition in melanoma models. Mol Cancer Ther. (2014) 13:2793–804. 10.1158/1535-7163.MCT-14-0452 25349308 PMC4263034

[15] Yadav V Zhang X Liu J Estrem S Li S Gong XQ. Reactivation of mitogen-activated protein kinase (MAPK) pathway by FGF receptor 3 (FGFR3)/Ras mediates resistance to vemurafenib in human B-RAF V600E mutant melanoma. J Biol Chem. (2012) 287:28087–98. 10.1074/jbc.M112.377218 22730329 PMC3431627

[16] Yadav V Burke TF Huber L Van Horn RD Zhang Y Buchanan SG. The CDK4/6 inhibitor LY2835219 overcomes vemurafenib resistance resulting from MAPK reactivation and cyclin D1 upregulation. Mol Cancer Ther. (2014) 13:2253–63. 10.1158/1535-7163.MCT-14-0257 25122067

[17] Jameson KL Mazur PK Zehnder AM Zhang J Zarnegar B Sage J. IQGAP1 scaffold-kinase interaction blockade selectively targets RAS-MAP kinase-driven tumors. Nat Med. (2013) 19:626–30. 10.1038/nm.3165 23603816 PMC4190012

[18] Wang J Chen J Miller DD Li W. Synergistic combination of novel tubulin inhibitor ABI-274 and vemurafenib overcome vemurafenib acquired resistance in BRAFV600E melanoma. Mol Cancer Ther. (2014) 13:16–26. 10.1158/1535-7163.MCT-13-0212 24249714 PMC3947172

[19] Ji Z Erin Chen Y Kumar R Taylor M Jenny Njauw CN Miao B. MITF Modulates Therapeutic Resistance through EGFR Signaling. J Invest Dermatol. (2015) 135:1863–72. 10.1038/jid.2015.105 25789707 PMC4466007

[20] Muller J Krijgsman O Tsoi J Robert L Hugo W Song C. Low MITF/AXL ratio predicts early resistance to multiple targeted drugs in melanoma. Nat Commun. (2014) 5:5712. 10.1038/ncomms6712 25502142 PMC4428333

[21] Sun X Li J Sun Y Zhang Y Dong L Shen C. miR-7 reverses the resistance to BRAFi in melanoma by targeting EGFR/IGF-1R/CRAF and inhibiting the MAPK and PI3K/AKT signaling pathways. Oncotarget. (2016) 7:53558–70. 10.18632/oncotarget.10669 27448964 PMC5288205

[22] Choi J Landrette SF Wang T Evans P Bacchiocchi A Bjornson R. Identification of PLX4032-resistance mechanisms and implications for novel RAF inhibitors. Pigment Cell Melanoma Res. (2014) 27:253–62. 10.1111/pcmr.12197 24283590 PMC4065135

[23] Das Thakur M Salangsang F Landman AS Sellers WR Pryer NK Levesque MP. Modelling vemurafenib resistance in melanoma reveals a strategy to forestall drug resistance. Nature. (2013) 494:251–5. 10.1038/nature11814 23302800 PMC3930354

[24] Basile KJ Abel EV Dadpey N Hartsough EJ Fortina P Aplin AE. *In vivo* MAPK reporting reveals the heterogeneity in tumoral selection of resistance to RAF inhibitors. Cancer Res. (2013) 73:7101–10. 10.1158/0008-5472.CAN-13-1628 24121492 PMC3851924

[25] Greger JG Eastman SD Zhang V Bleam MR Hughes AM Smitheman KN Combinations of BRAF, MEK, and PI3K/mTOR inhibitors overcome acquired resistance to the BRAF inhibitor GSK2118436 dabrafenib, mediated by NRAS or MEK mutations. Mol Cancer Ther. (2012) 11:909–20. 10.1158/1535-7163.MCT-11-0989 22389471

[26] Gowrishankar K Snoyman S Pupo GM Becker TM Kefford RF Rizos H. Acquired resistance to BRAF inhibition can confer cross-resistance to combined BRAF/MEK inhibition. J Invest Dermatol. (2012) 132:1850–9. 10.1038/jid.2012.63 22437314

[27] Atefi M von Euw E Attar N Ng C Chu C Guo D. Reversing melanoma cross-resistance to BRAF and MEK inhibitors by co-targeting the AKT/mTOR pathway. PLoS ONE. (2011) 6:e28973. 10.1371/journal.pone.0028973 22194965 PMC3237573

[28] Graziani G Artuso S De Luca A Muzi A Rotili D Scimeca M. A new water soluble MAPK activator exerts antitumor activity in melanoma cells resistant to the BRAF inhibitor vemurafenib. Biochem Pharmacol. (2015) 95:16–27. 10.1016/j.bcp.2015.03.004 25795251

[29] Su F Bradley WD Wang Q Yang H Xu L Higgins B. Resistance to selective BRAF inhibition can be mediated by modest upstream pathway activation. Cancer Res. (2012) 72:969–78. 10.1158/0008-5472.CAN-11-1875 22205714

[30] Atefi M Titz B Tsoi J Avramis E Le A Ng C. CRAF R391W is a melanoma driver oncogene. Sci Rep. (2016) 6:27454. 10.1038/srep27454 27273450 PMC4897636

[31] Kaplan FM Kugel CH III Dadpey N Shao Y Abel EV Aplin AE. SHOC2 and CRAF mediate ERK1/2 reactivation in mutant NRAS-mediated resistance to RAF inhibitor. J Biol Chem. (2012) 287:41797–807. 10.1074/jbc.M112.390906 23076151 PMC3516728

[32] Hu W Jin L Jiang CC Long GV Scolyer RA Wu Q. AEBP1 upregulation confers acquired resistance to BRAF (V600E) inhibition in melanoma. Cell Death Dis. (2013) 4:e914. 10.1038/cddis.2013.441 24201813 PMC3847319

[33] Fofaria NM Frederick DT Sullivan RJ Flaherty KT Srivastava SK. Overexpression of Mcl-1 confers resistance to BRAFV600E inhibitors alone and in combination with MEK1/2 inhibitors in melanoma. Oncotarget. (2015) 6:40535–56. 10.18632/oncotarget.5755 26497853 PMC4747351

[34] Thang ND Nghia PT Kumasaka MY Yajima I Kato M. Treatment of vemurafenib-resistant SKMEL-28 melanoma cells with paclitaxel. Asian Pac J Cancer Prev. (2015) 16:699–705. 10.7314/APJCP.2015.16.2.699 25684511

[35] Vergani E Vallacchi V Frigerio S Deho P Mondellini P Perego P. Identification of MET and SRC activation in melanoma cell lines showing primary resistance to PLX4032. Neoplasia. (2011) 13:1132–42. 10.1593/neo.111102 22241959 PMC3257188

[36] Anastas JN Kulikauskas RM Tamir T Rizos H Long GV von Euw EM. WNT5A enhances resistance of melanoma cells to targeted BRAF inhibitors. J Clin Invest. (2014) 124:2877–90. 10.1172/JCI70156 24865425 PMC4071371

[37] Obenauf AC Zou Y Ji AL Vanharanta S Shu W Shi H. Therapy-induced tumour secretomes promote resistance and tumour progression. Nature. (2015) 520:368–72. 10.1038/nature14336 25807485 PMC4507807

[38] Straussman R Morikawa T Shee K Barzily-Rokni M Qian ZR Du J. Tumour micro-environment elicits innate resistance to RAF inhibitors through HGF secretion. Nature. (2012) 487:500–4. 10.1038/nature11183 22763439 PMC3711467

[39] Parker R Vella LJ Xavier D Amirkhani A Parker J Cebon J. Phosphoproteomic analysis of cell-based resistance to BRAF inhibitor therapy in melanoma. Front Oncol. (2015) 5:95. 10.3389/fonc.2015.00095 26029660 PMC4432663

[40] Sharma R Fedorenko I Spence PT Sondak VK Smalley KS Koomen JM. Activity-based protein profiling shows heterogeneous signaling adaptations to BRAF inhibition. J Proteome Res. (2016) 15:4476–89. 10.1021/acs.jproteome.6b00613 27934295 PMC5642956

[41] Girotti MR Pedersen M Sanchez-Laorden B Viros A Turajlic S Niculescu-Duvaz D. Inhibiting EGF receptor or SRC family kinase signaling overcomes BRAF inhibitor resistance in melanoma. Cancer Discov. (2013) 3:158–67. 10.1158/2159-8290.CD-12-0386 23242808 PMC5321574

[42] Kim MH Kim J Hong H Lee SH Lee JK Jung E. Actin remodeling confers BRAF inhibitor resistance to melanoma cells through YAP/TAZ activation. EMBO J. (2016) 35:462–78. 10.15252/embj.201592081 26668268 PMC4772854

[43] Sandri S Faiao-Flores F Tiago M Pennacchi PC Massaro RR Alves-Fernandes DK. Vemurafenib resistance increases melanoma invasiveness and modulates the tumor microenvironment by MMP-2 upregulation. Pharmacol Res. (2016) 111:523–33. 10.1016/j.phrs.2016.07.017 27436149

[44] Faiao-Flores F Alves-Fernandes DK Pennacchi PC Sandri S Vicente AL Scapulatempo-Neto C. Targeting the hedgehog transcription factors GLI1 and GLI2 restores sensitivity to vemurafenib-resistant human melanoma cells. Oncogene. (2017) 36:1849–61. 10.1038/onc.2016.348 27748762 PMC5378933

[45] Cordaro FG De Presbiteris AL Camerlingo R Mozzillo N Pirozzi G Cavalcanti E. Phenotype characterization of human melanoma cells resistant to dabrafenib. Oncol Rep. (2017) 38:2741–51. 10.3892/or.2017.5963 29048639 PMC5780027

[46] Caporali S Alvino E Lacal PM Levati L Giurato G Memoli D. Targeting the PI3K/AKT/mTOR pathway overcomes the stimulating effect of dabrafenib on the invasive behavior of melanoma cells with acquired resistance to the BRAF inhibitor. Int J Oncol. (2016) 49:1164–74. 10.3892/ijo.2016.3594 27572607

[47] Ross KC Andrews AJ Marion CD Yen TJ Bhattacharjee V. Identification of the serine biosynthesis pathway as a critical component of BRAF inhibitor resistance of melanoma, pancreatic, and non-small cell lung cancer cells. Mol Cancer Ther. (2017) 16:1596–609. 10.1158/1535-7163.MCT-16-0798 28500236 PMC5544579

[48] Hernandez-Davies JE Tran TQ Reid MA Rosales KR Lowman XH Pan M. Vemurafenib resistance reprograms melanoma cells towards glutamine dependence. J Transl Med. (2015) 13:210,015-0581-2. 10.1186/s12967-015-0581-2 26139106 PMC4490757

[49] McDermott M Eustace AJ Busschots S Breen L Crown J Clynes M. *in vitro* development of chemotherapy and targeted therapy drug-resistant cancer cell lines: a practical guide with case studies. Front Oncol. (2014) 4:40. 10.3389/fonc.2014.00040 24639951 PMC3944788

[50] Zeiderman MR Egger ME Kimbrough CW England CG Dupre TV McMasters KM. Targeting of BRAF resistant melanoma via extracellular matrix metalloproteinase inducer receptor. J Surg Res. (2014) 190:111–8. 10.1016/j.jss.2014.02.021 24655664 PMC4576881

[51] Tirosh I Izar B Prakadan SM Wadsworth MH II Treacy D Trombetta JJ. Dissecting the multicellular ecosystem of metastatic melanoma by single-cell RNA-seq. Science. (2016) 352:189–96. 10.1126/science.aad0501 27124452 PMC4944528

[52] Domcke S Sinha R Levine DA Sander C Schultz N. Evaluating cell lines as tumour models by comparison of genomic profiles. Nat Commun. (2013) 4:2126. 10.1038/ncomms3126 23839242 PMC3715866

[53] Jiang G Zhang S Yazdanparast A Li M Pawar AV Liu Y. Comprehensive comparison of molecular portraits between cell lines and tumors in breast cancer. BMC Genomics. (2016) 17(Suppl. 7):525,016-2911-z. 10.1186/s12864-016-2911-z 27556158 PMC5001206

[54] Sinha R Winer AG Chevinsky M Jakubowski C Chen YB Dong Y. Analysis of renal cancer cell lines from two major resources enables genomics-guided cell line selection. Nat Commun. (2017) 8:15165. 10.1038/ncomms15165 28489074 PMC5436135

[55] Vincent KM Findlay SD Postovit LM. Assessing breast cancer cell lines as tumour models by comparison of mRNA expression profiles. Breast Cancer Res. (2015) 17:114,015-0613-0. 10.1186/s13058-015-0613-0 26289960 PMC4545915

[56] Vincent KM Postovit LM. Investigating the utility of human melanoma cell lines as tumour models. Oncotarget. (2017) 8:10498–509. 10.18632/oncotarget.14443 28060736 PMC5354675

[57] Grippo JF Zhang W Heinzmann D Yang KH Wong J Joe AK. A phase I, randomized, open-label study of the multiple-dose pharmacokinetics of vemurafenib in patients with BRAF V600E mutation-positive metastatic melanoma. Cancer Chemother Pharmacol. (2014) 73:103–11. 10.1007/s00280-013-2324-5 24178368

[58] Falchook GS Long GV Kurzrock R Kim KB Arkenau HT Brown MP. Dose selection, pharmacokinetics, and pharmacodynamics of BRAF inhibitor dabrafenib (GSK2118436). Clin Cancer Res. (2014) 20:4449–58. 10.1158/1078-0432.CCR-14-0887 24958809

[59] Funck-Brentano E Alvarez JC Longvert C Abe E Beauchet A Funck-Brentano C. Plasma vemurafenib concentrations in advanced BRAFV600mut melanoma patients: impact on tumour response and tolerance. Ann Oncol. (2015) 26:1470–5. 10.1093/annonc/mdv189 25899783

[60] Tap WD Gong KW Dering J Tseng Y Ginther C Pauletti G. Pharmacodynamic characterization of the efficacy signals due to selective BRAF inhibition with PLX4032 in malignant melanoma. Neoplasia. (2010) 12:637–49. 10.1593/neo.10414 20689758 PMC2915408

[61] He Y Zhu Q Chen M Huang Q Wang W Li Q. The changing 50% inhibitory concentration (IC50) of cisplatin: a pilot study on the artifacts of the MTT assay and the precise measurement of density-dependent chemoresistance in ovarian cancer. Oncotarget. (2016) 7:70803–21. 10.18632/oncotarget.12223 27683123 PMC5342590

[62] Clark NA Hafner M Kouril M Williams EH Muhlich JL Pilarczyk M. GRcalculator: an online tool for calculating and mining dose-response data. BMC Cancer. (2017) 17:3689–3. 10.1186/s12885-017-3689-3 29065900 PMC5655815

